# Increasing student engagement using an Amazing Race–style competition

**DOI:** 10.5195/jmla.2021.1178

**Published:** 2021-07-01

**Authors:** Emily F. Gorman

**Affiliations:** 1 efgorman@hshsl.umaryland.edu, University of Maryland, Baltimore, Baltimore, MD

**Keywords:** game-based learning, drug information resources, library instruction

## Abstract

**Background:**

Game-based learning is a successful strategy for teaching various concepts to students, from general orientations to more in-depth material. Pharmacy students in a first-year lab course were introduced to library and drug information resources through a lecture-style class in their first week of school, which was ineffective in terms of engagement. To combat this issue, the pharmacy liaison librarian advocated for moving this class session later in the semester and proposed a game-based activity to replace the lecture.

**Case Presentation:**

“The Amazing Race: Drug Information Edition” was inspired by a well-known TV competition that involves completing several stages (called “legs”) of challenges to finish the race. The librarian developed questions designed to make students use various parts of the library website as well as two drug information databases. Students competed in teams, and the first three teams to complete the race were awarded small prizes. The race was first implemented in 2018, and modifications were made to the 2019 iteration based on student feedback.

**Conclusions:**

Despite several challenges, the race was well received by both the students and the course instructors and increased engagement with introductory library and drug information material. The activity has enhanced the librarian's relationship and collaboration with the course faculty and made a positive impression on the students.

## BACKGROUND

Instructors are constantly seeking ways to enhance student engagement in classroom learning, and introducing gaming principles into activities has been one effective strategy. Librarians have successfully used games for orientations to library resources and services [1, 2] as well as to teach more complex concepts such as information literacy [3–6] and evidence-based medicine [7–9]. Similar strategies are being employed in health sciences curricula as well. Pharmacy students have participated in games to learn about proper medication history-taking [10] and diabetes treatment [11], and games have helped medical students reinforce their foundational physiology knowledge [12].

Librarians and other instructors have developed a wide variety of games, based on everything from board games [13] to the more recently popular escape rooms [1, 11]. Another source of inspiration has been well-known television shows. Successful learning games have been based on *The Amazing Race* [6, 14], *Who Wants to Be a Millionaire?* [12], and *Survivor* [9], among others. The author utilized this approach in an effort to increase student engagement in an introductory library and drug information resources class with first-year pharmacy students. This paper describes the development and implementation of “The Amazing Race: Drug Information Edition” over two years, as well as the feedback it received from students.

Although there is published research about game-based learning in pharmacy, there is a paucity of literature about librarian instruction in pharmacy laboratory courses. The author found one article about a librarian collaborating with a pharmacist in a laboratory class, but it involved second-year students and did not incorporate gamification [15]. The setting of this case report, game-based instruction by a librarian in a first-year pharmacy laboratory course, appears to be unique in the literature to date.

## CASE PRESENTATION

### Course context

PHAR 505: Abilities Lab 1 (ABL 1) is the first course in a three-year laboratory series that teaches pharmacy-related skills to students. These skills include communication, drug information (DI), patient education, IV preparation, and vital signs measurement. When the author first began working with this course, first-year students were introduced to library and DI resources during a lecture on the first day of class and another lecture later in the semester. Although these lectures did involve an in-class worksheet exercise, the classes were not very engaging. Both involved a discussion of material that students tend to find dry—database demonstrations and citation guidelines—followed by a simple worksheet. That format was not enough to significantly engage students on the first day of class, when they were overwhelmed with all the new information they were receiving during the first week in the PharmD program.

In an effort to make the orientation to library and DI resources more engaging, the first lecture was replaced with a game activity inspired by the popular TV competition *The Amazing Race* [16]. On this show, contestants are racing to complete a series of challenges within stages of the race, referred to as “legs.” The teams must finish one leg of the race before receiving the clues for the next leg.

### Amazing Race—fall 2018

The author adapted an Amazing Race orientation activity from her previous institution to fit the current context. “The Amazing Race: Drug Information Edition” involved five legs, each with a series of questions that prompted students to use specific library resources to find the answers. The legs included Library Services, Pharmacy Subject Guide, OneSearch (the library's discovery service), Micromedex, and Lexicomp. Each leg consisted of four questions, which were tested with library colleagues prior to implementation. Each set of questions is independent from the others, so the legs can be used in any order.

The first implementation of the Amazing Race game was in fall 2018. The class of 132 students was split in half to make the activity more manageable. The game was run twice, back-to-back, with 2 groups of 66 students. The author led the game, with support from 2 course instructors and approximately 6 fourth-year pharmacy students who were on their teaching rotation.

Prior to class, students were instructed to watch a brief introductory video on the basics of using the library website, Micromedex, and Lexicomp. In class, the author introduced the Amazing Race activity and reviewed the instructions, then supervised and answered questions during the race. Students competed in self-selected teams of four and were allowed to come up with their own team names for the competition (after being reminded to keep things professional). Everyone received an answer sheet, which each team member had to fill out individually. Teams were restricted to using two computing devices to ensure all members were engaging with each question rather than just dividing up the questions.

When a team finished answering all the questions for one leg, their team captain brought all the answer sheets to a facilitator to be checked. Facilitators checked the answers against their answer keys, and if everything was correct, the team captain was given the clue sheet for the next leg of the race. The first three teams to finish the race were given small prizes of candy and library-branded pens and highlighters, but all teams were expected to finish the activity even after these winning spots were taken. At the end, students completed an online evaluation form (see Data Availability Statement). The activity, including the time to introduce the rules and distribute the materials, took about forty minutes, although the time it took individual teams to complete the race varied.

Overall, the race went as smoothly as it could have for the first iteration. Considering the number of students involved, a few speedbumps were inevitable. It was discovered during the race that there were several correct answer options that had not been included on the answer keys, and there were a few questions that were unclear to several teams. One leg of the race was about the Pharmacy Subject Guide (a LibGuide), and the instructions for that section confused many of the students. Also, several students complained in the course evaluations that the teams of four were only allowed to use two computers. That restriction had been imposed out of a concern that, if every student on a team used their own device, the teams would just split up the questions and each student would only be exposed to one question per leg. However, one of the complaints mentioned that it made two of the team members feel left out or that they could not contribute, which was a valid concern.

### Amazing Race—fall 2019

**Figure 1:**
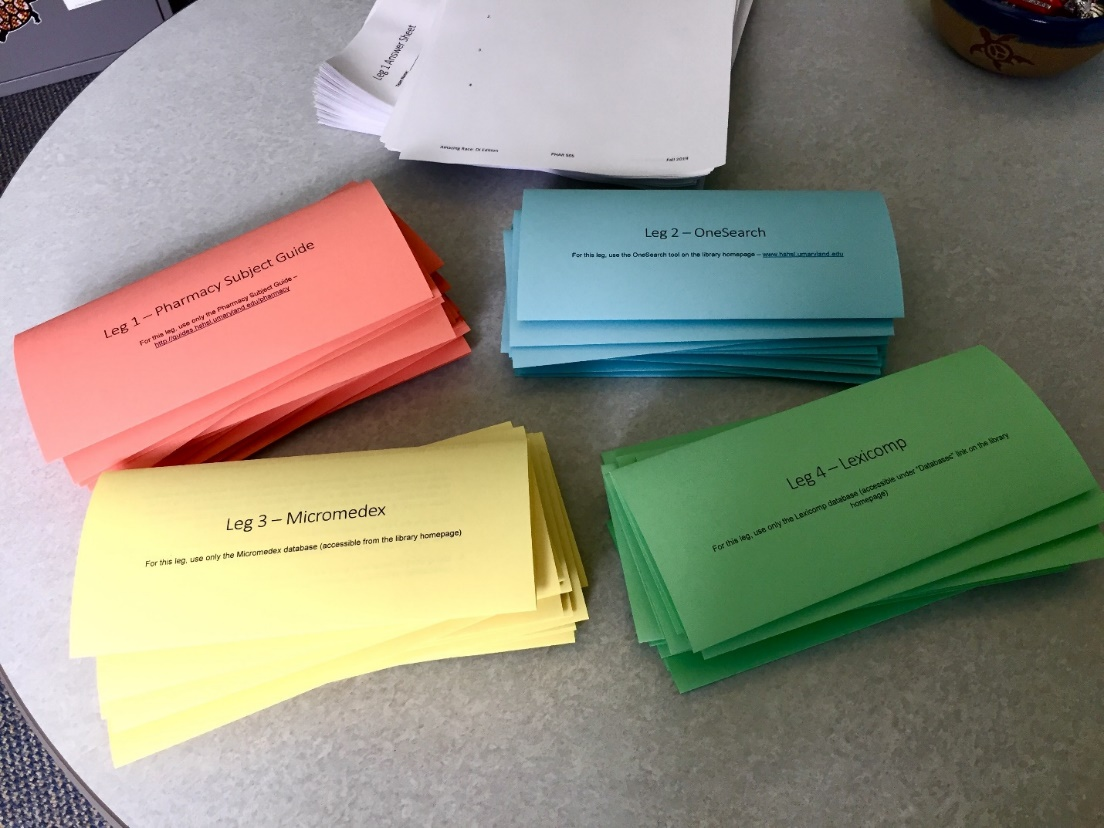
Amazing Race clue and answer sheets for fall 2019

Based on student feedback, a number of changes were made to the Amazing Race for its second iteration in fall 2019. Students indicated that the Library Services leg did not provide useful information, so it was removed. This leg included very basic library information such as opening hours and where to find the workshop schedule, which was included in the introductory video student were supposed to watch before class. The evaluations also suggested that the students wanted more background on drug information before jumping into the activity. Rather than replace the Library Services leg, the race was reduced to four legs so that there was more time to add a brief introductory presentation about the author's role, the class's learning objectives, and the concept of drug information (Figure 1).

Despite being tested on coworkers beforehand, some questions in the first iteration of the game had caused confusion and necessitated on-the-spot additions of more correct answers to the answer keys. These questions were changed for the second iteration, and the Pharmacy Subject Guide leg was reworked so that it did not need the instructions that students found confusing. Additionally, the restriction on the number of computers per team was removed based on the complaints from the first iteration. While there was still a concern about students not being involved in answering all the questions, it was hoped that the requirement for each team member to complete their own individual answer sheet would mitigate that problem. Instructions and answer keys for both iterations of the race are available in the supplementary material.

Overall, the student evaluations were positive, and the changes made for 2019 seemed to be successful in addressing some of the problems from the first implementation. In fall 2018, 112 students completed the evaluation; 82 completed it in fall 2019. Most students in both years (93.6% in 2018, 95.1% in 2019) indicated that they learned something new, and most (74.8% in 2018, 81.7% in 2019) also rated the activity as effective.

The majority of the negative comments consisted of students saying they would not retain the material because of the fast pace of the activity. However, other students responded that the competitive race made them enjoy the activity. Ultimately, there were more positive comments than negative ones (see Table 1). Retention of the material from this activity was not a major concern, since the students were receiving further DI instruction in two subsequent class sessions. The main goal of the race was to make a boring library orientation session more fun and engaging so students would remember the library and their librarian. However, the author is continuing to consider ways to keep the activity competitive while slowing it down a bit so it is less stressful and students pay more attention to the information.

**Table 1:** Selection of student comments from the activity evaluation survey

Comment	Year
“I loved the exercise! I learned a lot! Thank you!”	2019
“This was a very practical and effective exercise! I usually don't races like this but I guess if it's kept rare like this occurance [*sic*] then it can be a fun change of pace.”	2019
“Neutral; the unnecessary pressure of the class activity inhibits comprehensive learning of the databases. It would have been better had the pace of the activity was more relaxed (better material retention)”	2019
“The amazing race competition made the activity fun and competitive”	2018
“I liked the activity! It was a fun way to access information in the library website.”	2018

## DISCUSSION

This activity has been very beneficial in terms of introducing the librarian to the pharmacy students in a positive and fun way. After the first implementation, the author overheard students in the hall after class remarking how fun the activity was, and there is anecdotal evidence for its lasting positive impression as well. In a recent meeting with instructors and student facilitators regarding the fall 2020 lab course, one student still remembered her team winning the Amazing Race two years earlier. This positive reaction is similar to the experience described by previous authors who have implemented an Amazing Race activity [6].

Another strength of this activity was its engagement of the fourth-year pharmacy students in their teaching rotation. Participating as facilitators of the race gave these students a unique teaching experience compared to other types of instruction they were exposed to during their time in the lab course. They were also instrumental in providing the librarian with feedback about which questions were causing problems during the activity.

In addition to benefiting the students, the activity has also improved faculty relationships. The managers of the lab course love the race, so it has enhanced the author's rapport with them and enabled closer collaboration. In fact, the activity has gained national recognition by being selected as the winner of a teaching innovation award from a pharmacy professional organization. That award has also helped raise the author's profile as a peer and instructor among the pharmacy faculty.

There were several challenges with this activity, and the author continues to learn from mistakes in each iteration. One challenge was trying to create questions that had a limited number of correct answers and ways to find the answers. Some of the students managed to find pages and links the author did not know were on the library website, which illustrates that even if one thinks that all possible ways to answer a question have been identified, one must be prepared to accommodate new answers during the activity. Testing the questions on coworkers does not identify all potential problems, because library workers likely use resources in a different way than students. Student workers, if available, might be a better test group.

Another challenge was that the students finished the race at different times; some teams finished quite quickly, while others struggled and were not able to finish in the time available. Teams that finished quickly were left waiting after filling out the class evaluation, while the teams that struggled lacked motivation to finish the race once the top three spots with prizes were taken. Consider having some sort of task or reading for students to do if they finish the race early.

Lastly, the feedback from the first implementation of the race indicated that it is helpful to provide some background and context before jumping into the activity. Students were supposed to watch an introductory video about the library website and drug information databases before coming to lab, but those who did not watch it were unprepared and felt the activity was coming out of nowhere. In the second year of the race, a brief presentation about drug information was added to the beginning of the class before starting the activity so students would understand why questions about how to use the drug information databases were included in the race.

Overall, the benefits of the activity have outweighed the challenges, and the author plans to continue to implement it. Due to virtual instruction necessitated by the COVID-19 pandemic, the race was not run in fall 2020; instead, it was modified into an online escape room activity. However, once in-person instruction resumes, the race can continue.

## Data Availability

Data associated with this article are available in the UMB Digital Archive at http://hdl.handle.net/10713/14099 and http://hdl.handle.net/10713/14103.

## SUPPLEMENTAL FILES

**Supplemental File 1:** PHAR 505 Amazing Race Evaluation 2019

**Supplemental File 2:** Amazing Race: Drug Information Edition instructions and answer keys
