# Supplementary material for: Increasing student engagement using an Amazing Race–style competition
Source: J Med Libr Assoc. 2021 Jul 1;109(3):478–82. doi: 10.5195/jmla.2021.1178 (PMC8485938; doi:10.5195/jmla.2021.1178)
Supplement: Supplementary file 1 — Supplemental File 1: PHAR 505 Amazing Race Evaluation 2019 [file jmla-109-3-478-s01.pdf]

PHAR 505 Amazing Race Evaluation 2019

Start of Block: Default Question Block

Q1 What drug information resources were you familiar with prior to today's class?

- [ ] Clinical Pharmacology (1)
- [ ] Epocrates (2)
- [ ] Facts and Comparisons (3)
- [ ] Lexicomp (4)
- [ ] Micromedex (5)
- [ ] Print books (6)
- [ ] None (7)
- [ ] Other (please specify) (8) ____

Q2 Did you learn something new during the Amazing Race activity?

- ○ Yes (1)
- ○ No (2)

Q3 What information covered today was most useful?

____

____

Q4 What information covered today was least useful?

Q5 Are there any topics you wish had been covered in more detail in today's class?

Q6 Rate the overall effectiveness of today's class (content, activities, pacing, etc.).

	Poor (1)	Adequate (2)	Neutral (3)	Good (4)	Excellent (5)
Overall effectiveness	○	○	○	○	○

Q7 Use this space for any additional comments, including suggestions for improving the class.

____

____

____

____

____

End of Block: Default Question Block
