# Supplementary material for: Increasing student engagement using an Amazing Race–style competition
Source: J Med Libr Assoc. 2021 Jul 1;109(3):478–82. doi: 10.5195/jmla.2021.1178 (PMC8485938; doi:10.5195/jmla.2021.1178)
Supplement: Supplementary file 2 — Supplemental File 2: Amazing Race: Drug Information Edition instructions and answer keys [file jmla-109-3-478-s02.docx]

Supplementary Materials to Accompany “Increasing Student Engagement Using an Amazing Race-Style Competition” by Emily F. Gorman

# Fall 2018 Amazing Race: Drug Information Edition

Instructions:

1. Split into groups of 4 people.
2. Come up with a team name and choose a team captain.
3. Send your captain down to the podium to write your team’s name on the screen and collect answer sheets for your team.
4. Write your team’s name at the top of your answer sheets for all five (5) legs of the race.

Rules:

- A maximum of two (2) computing devices (smartphones, laptops, tablets, etc.) can be used per team. If we see more than two being used your team will be disqualified from winning.
- There are 5 legs with 4 questions each. For each leg, you are only allowed to use the specified resource to answer the questions.
- Every team member must complete an answer sheet for each leg and your team captain must bring all answer sheets to an instructor for scoring. **Every team member’s answer sheet must be filled out correctly before your team can receive the next set of clues.**
- The first three teams to complete all five legs of the race will win a prize!

### Leg 1 – Library Services

For this leg, use only the library website – [www.hshsl.umaryland.edu](http://www.hshsl.umaryland.edu)

1. Where can you find the Research Consultation service?
   1. Under the assistance tab OR under “Help with Your Research”
2. What are the library’s regular building hours (full week)?
   1. Mon-Thurs 6am-1am, Fri 6am-8pm, Sat 8am-8pm, Sun 8am-10pm
3. What are three ways you can contact the library for assistance?
   1. Chat, submit a question, email, call/phone, text message, in person at desk (any 3 are acceptable)
4. What is the date, time, and room number of the library workshop called “Advanced Searching in PubMed and Embase”?
   1. November 13, 12pm, Room LL03

### Leg 2 – Pharmacy Subject Guide

For this leg, use only the Pharmacy Subject Guide - <http://guides.hshsl.umaryland.edu/pharmacy>

For each question, list the name of the tab/page that contains the specified resource:

1. Pharmacy Librarian’s (Emily Gorman’s) contact information
   1. Getting Started
2. Orange Book
   1. Finding Drug and Clinical Information
3. MedlinePlus
   1. Patient Education Resources
4. Natural Medicines
   1. Complementary and Alternative Medicine OR Finding Drug and Clinical Information

### Leg 3 – OneSearch

For this leg, use the OneSearch tool on the library homepage – [www.hshsl.umaryland.edu](http://www.hshsl.umaryland.edu)

1. What is the location and call number of the *Handbook of Nonprescription Drugs*?
   1. 5^th^ floor stacks OR Reference Collection, RM671.A1 H34
2. In what formats is *Harrison’s Principles of Internal Medicine* available?
   1. Print and electronic (eBook is also okay)
3. List **all** of the locations where the **2014 edition** of *Pharmacotherapy: A Pathophysiologic Approach* (edited by DiPiro) is available.
   1. Health Sciences Reserve Desk, Loyola Notre Dame Library Health Science Reference – 3^rd^ level, Shady Grove Library Course Reserves
4. How many copies of the 2016 edition of Goldman-Cecil Medicine are available at the HS/HSL?
   1. 2

### Leg 4 – Micromedex

For this leg, use only the Micromedex database (accessible from the library homepage).

1. EB has a history of motion sickness and is planning to use **scopolamine** patches on an upcoming cruise with her family. She wants to make sure it is okay for her to use this medication, as she also takes **amlodipine** for hypertension, **bupropion** for depression, and has a **sulfa allergy**. Are there interactions between any of these drugs? If so, list the drugs and the severity of the interaction.
   1. Scopolamine and bupropion hydrochloride, Major (also okay if they say bupropion and leave out the hydrochloride)
2. What is the average wholesale price per unit (AWP Unit Price) of a 9.9ml package of Flonase (hint: use the RED BOOK tool)?
   1. 1.43535 (also okay if they round this to 1.44 or similar)
3. How many languages is the CareNotes DrugNote on regular insulin available in?
   1. 15
4. Using Drug Comparison, compare Zyrtec (cetirizine hydrochloride) and Claritin (loratidine). Which drug has more FDA-approved uses (indications)?
   1. Zyrtec OR cetirizine hydrochloride

### Leg 5 – Lexicomp

For this leg, use only the Lexicomp database (accessible under “Databases” link on the library homepage).

1. An unidentified peach oblong tablet has 018 imprinted on one side. What drug is it (generic name)?
   1. Fexofenadine
2. DK takes atomoxetine (Strattera) to treat her ADHD and recently found out she is pregnant. She wants to know whether it is safe for her to continue taking this medication during her pregnancy. Locate the *Briggs Drugs in Pregnancy and Lactation* monograph for atomoxetine. What is the pregnancy **recommendation**?
   1. Limited human data, animal data suggest risk
3. Look up the IV compatibility of Benadryl (diphenhydramine hydrochloride), lorazepam, and ampicillin sodium. Which pair of drugs is compatible in the Y-Site but **not** as an admixture?
   1. Benadryl and lorazepam
4. Is the patient education leaflet for regular insulin (adult) available in Greek?
   1. Yes

# Fall 2019 Amazing Race: Drug Information Edition

Instructions:

1. Split into groups of 4 people.
2. Come up with a team name and choose a team captain.
3. Send your captain down to the podium to write your team’s name on the screen and collect answer sheets for your team.
4. Write your team’s name at the top of your answer sheets for all five (5) legs of the race.

Rules:

- There are 4 legs with 4 questions each. For each leg, you are only allowed to use the specified resource to answer the questions.
- Every team member must complete an answer sheet for each leg and your team captain must bring all answer sheets to an instructor for scoring. **Every team member’s answer sheet must be filled out correctly before your team can receive the next set of clues.**
- The first three teams to complete all four legs of the race will win a prize!

### Leg 1 – Pharmacy Subject Guide

For this leg, use only the Pharmacy Subject Guide - <http://guides.hshsl.umaryland.edu/pharmacy>

1. List **two** **mobile resources** that are available for finding drug and clinical information. **(any 2 are acceptable)**
   1. Micromedex Drug Information, Drug Interactions, and IV Compatibility
   2. Epocrates
   3. LactMed App
   4. Drug Information Portal Mobile
   5. TOXNET Mobile
   6. Orange Book Express
   7. About Herbs
2. List **two** sources of patient-friendly health information. **(any 2 are acceptable)**
   1. MedLine Plus
   2. Understanding Prescription Medication Labels
   3. About Herbs
   4. HealthReach
   5. Micromedex
   6. Lexicomp Online (just Lexicomp is also acceptable)
   7. UpToDate
3. List **three** databases you could use to find scholarly journal articles. **(any 3 are acceptable)**
   1. PubMed
   2. Embase
   3. International Pharmaceutical Abstracts
   4. Cochrane Library
   5. SciFinder
   6. Scopus
4. Name a resource you could use to find information on complementary and alternative medicine. **(any 1 is acceptable)**
   1. Cancer.gov—Complementary and Alternative Medicine
   2. FDA: Dietary Supplements
   3. IBIDS Database
   4. MedlinePlus: Complementary and Alternative Medicine
   5. Center for Integrative Medicine (CIM) at University of Maryland School of Medicine
   6. Memorial Sloan-Kettering Cancer Center (MSKCC) – About Herbs, Botanicals & Other Products
   7. NCCIH: National Center for Complementary and Integrative Health
   8. Quackwatch
   9. Natural Medicines
   10. CAM on PubMed
   11. Cochrane Complementary Medicine
   12. Complementary medicine in clinical practice : integrative practice in American healthcare
   13. Mosby's complementary & alternative medicine : a research-based approach
   14. Natural alternative and complimentary health care practices
   15. Textbook of complementary and alternative medicine
   16. The ACP evidence-based guide to complementary & alternative medicine
   17. WHO global atlas of traditional, complementary and alternative medicine
   18. BMC Complementary and Alternative Medicine
   19. Evidence-based Complementary & Alternative Medicine
   20. Natural Health

### Leg 2 – OneSearch

For this leg, use the OneSearch tool on the library homepage – [www.hshsl.umaryland.edu](http://www.hshsl.umaryland.edu)

1. What is the **HS/HSL** location and call number of the **2018 edition** of the *Harriet Lane Handbook*?
   1. Health Sciences Reserve Desk, RJ48 .H35 2018
2. In what formats is *Harrison’s Principles of Internal Medicine* available?
   1. Print and electronic (eBook is also okay)
3. List **all** of the locations where the **2014 edition** of *Pharmacotherapy: A Pathophysiologic Approach* (edited by DiPiro) is available.
   1. Health Sciences 5^th^ floor stacks, Shady Grove Library Course Reserves
4. How many copies of the **2013 edition** of *Applied Therapeutics: The Clinical Use of Drugs* are available at the HS/HSL?
   1. 3

### Leg 3 – Micromedex

For this leg, use only the Micromedex database (accessible from the library homepage).

1. EB has a history of motion sickness and is planning to use **scopolamine** patches on an upcoming cruise with her family. She wants to make sure it is okay for her to use this medication, as she also takes **lisinopril** for hypertension, **bupropion** for depression, and has a **sulfa allergy**. Are there interactions between any of these drugs? If so, list the drugs and the severity of the interaction.
   1. Scopolamine and bupropion hydrochloride, Major (also okay if they say bupropion and leave out the hydrochloride)
2. What are the FDA approved indications (uses) for alprazolam (Xanax)?
   1. Anxiety and Panic Disorder
3. How many languages is the CareNotes DrugNote on regular insulin available in?
   1. 15
4. Using Drug Comparison, compare Zyrtec (cetirizine hydrochloride) and Claritin (loratadine). Which drug has more adverse effects?
   1. Zyrtec (cetirizine hydrochloride is also acceptable)

### Leg 4 – Lexicomp

For this leg, use only the Lexicomp database (accessible under “Databases” link on the library homepage).

1. An unidentified peach oblong tablet has 018 imprinted on one side. What drug is it (generic name)?
   1. Fexofenadine
2. DK takes atomoxetine (Strattera) to treat her ADHD and recently found out she is pregnant. She wants to know whether it is safe for her to continue taking this medication during her pregnancy. Locate the *Briggs Drugs in Pregnancy and Lactation* monograph for atomoxetine. What does it say under **Pregnancy** **Recommendation**?
   1. Limited human data, animal data suggest risk
3. Look up the IV compatibility of Benadryl (diphenhydramine hydrochloride), lorazepam, and ampicillin sodium. Which pair of drugs is compatible in the Y-Site? **(brand or generic names acceptable)**
   1. Benadryl and lorazepam
4. What is the **geriatric** dosing of immediate release clonidine?
   1. Initial 0.1 mg once daily at bedtime, increase gradually as needed
